# A matrix approach to visually communicate simultaneously the environmental and health impacts of foods

**DOI:** 10.3389/fnut.2025.1572297

**Published:** 2025-08-05

**Authors:** Andrew Berardy, Ujué Fresán, Nazanin Abbaspour, Joan Sabaté

**Affiliations:** ^1^Center for Nutrition, Lifestyle and Disease Prevention, School of Public Health, Loma Linda University, Loma Linda, CA, United States; ^2^Department of Geography and Environmental Engineering, United States Military Academy, West Point, NY, United States; ^3^e-Health Group, ISGlobal, Barcelona, Spain

**Keywords:** environmental sustainability, human health, food groups, relative risk, health index

## Abstract

**Introduction:**

Despite an unprecedented wealth of knowledge regarding the environmental and health effects of foods, no studies effectively and simultaneously communicate both characteristics in an easily comprehensible visual format. This work, therefore aims to provide a clear visualization that intuitively demonstrates the relative characteristics of a comprehensive list of foods to encourage more informed decision-making across stakeholders.

**Methods:**

Data are aggregated from meta-analyses and reviews regarding the carbon footprint (CFP) and health effects of 30 food groups commonly consumed in the United States of America. The data are then used to categorize food groups as favorable, neutral, or unfavorable to health and as having a low, medium, or high carbon footprint. These classifications are then used to arrange food groups into a three by three color-coded matrix.

**Results:**

The resulting visualization simultaneously communicates environmental impacts and health implications of food groups in a single figure. Overall, the visualization indicates plant-based and less processed foods are preferable to animal-based and more processed foods. An exception to the format is made to emphasize the exceptionally large carbon footprint of beef by splitting the lower-right cell into two halves.

**Discussion:**

Classification of food groups according to health effects and carbon footprint is consistent with results presented in other studies. The color-coded matrix format quickly and intuitively communicates the tradeoffs made when choosing between different food groups, which may help improve choices for human and planetary health.

## 1 Introduction

Production and consumption of food has a significant impact on the environment and human health. Agriculture is the largest driver of global environmental change, including emitting 30% of greenhouse gasses, using 70% of freshwater, and occupying 40% of global land surface to meet global demands for food and modern diets high in animal products and ultra processed foods, with conversion of natural ecosystems to cropland and pastures as the largest factor threatening species with extinction, while current dietary patterns are the leading cause of morbidity and mortality worldwide (1). Lack of whole grains and fruits and excessive intake of sodium account for more than 50% of deaths and 66% of disability adjusted life years globally (2). Therefore, it is of urgent importance to reduce these negative impacts of the current food system.

Potential strategies for reducing the environmental impacts caused by the food system include improving agricultural technologies to increase efficiency of production, reducing food loss and food waste to lower production needs, and promoting the production and consumption of foods that are better for human and planetary health (3–6). The type and amount of food produced and consumed are major determining factors for promoting human health within planetary boundaries (7, 8).

Some countries include environmental sustainability in their dietary guidelines and food policies to encourage both health and environmental improvement (9–13). However, broader systematic changes are required at all levels including increasing access and affordability and changing consumer behavior to achieve a sustainable food system. Economic and socio-cultural factors must also be considered when making such changes (14). Nevertheless, even small dietary adjustments can significantly improve environmental and health outcomes when based on well-targeted recommendations (15). To be effective, such recommendations must effectively communicate potential tradeoffs and synergies between human health and the environment.

The impact of global dietary trends on both human health and environmental sustainability is a subject of significant interest in recent years (3, 4, 16, 17). Studies are conducted to better understand this relationship, breaking down the effects of diet into various dietary types (18, 19), food groups (20), and even individual food products found in supermarkets (21). The results consistently show that consuming a diet rich in whole plant foods benefits both human health and the environment (13, 22, 23). Nevertheless, there is a need for a comprehensive yet easy-to-understand presentation of these findings to reach a broader audience (24, 25). Existing literature examines the health and environmental implications of foods but does not provide a visualization sufficiently simple and comprehensive for a broad audience to intuitively understand tradeoffs between most commonly consumed food choices (17, 20, 21).

The purpose of this manuscript is to introduce an evidence-based visual aid that effectively communicates both the health and sustainability implications of food choices. Therefore, this article introduces a clear and concise visualization that simultaneously represents the health and environmental impacts of 30 food groups commonly consumed in the United States of America (USA).

## 2 Materials and methods

### 2.1 Visualization design

Visualization and classification of foods across two dimensions is well-suited for display in a matrix format, with each direction representing a change in one of the two dimensions. A three-by-three matrix allows for distinction between the worst and best performing options as well as those in the middle, balancing simplicity and comprehensiveness. Environmental performance is represented by using carbon footprints (CFP) while health performance is represented by using a calculated Health Index Score (HIS), based on meta-analyses and reviews reporting the relative risk of various health outcomes.

### 2.2 Selection and classification of food groups

The matrix includes 30 food groups chosen through consultation with nutrition professionals with the goal of identifying representative categories for the most commonly consumed food groups in the USA that balance inclusiveness and simplicity. For this purpose, similar foods are grouped together using straightforward names, e.g., all tubers are included in the group “potatoes.” Table 1 provides a list of the food groups and their constituent foods.

**Table 1:** Food groups list.

**Food groups**	**Description**
Fruits	All fruits, fresh, frozen, canned, or dried
Vegetables	All vegetables, except tubers and legumes, fresh, frozen, canned, or dried
Potatoes	All potatoes and other tubers, boiled, baked, fried but not processed chips or snacks (e.g., potato chips)
Beans and peas	All legumes, fresh, cooked, frozen, or canned
Nuts and seeds	All nuts and seeds, natural, roasted, salted
Whole grains	All whole grains, including bread and milled flour products
Refined grains	All refined grains, including bread and milled flour products
Ready-to-eat cereals	All commercial breakfast cereals. It does not include home-made granola or muesli
Pastries and desserts	All desserts based on flour or milk and high in added sugars and fats
Savory snacks	All processed snacks, including chips and pretzels
Fish	All types of fish, fresh, frozen, or canned
Shellfish	Crustaceans (e.g., lobster, prawn, or shrimp)
Poultry	Unprocessed poultry such as chicken, turkey
Processed meats	Processed red or white meat such as ham, bacon, or sausages
Beef	Unprocessed meat from ruminants, including beef, lamb, and
Pork	Unprocessed meat from pigs
Eggs	Boiled, fried, scrambled, or in dishes
Dairy products	Any milk, yogurt, cheese
Dairy substitutes	Plant-based products that mimic dairy
Meat substitutes	Plant-based protein products, including tofu
Vegetable oils	Any liquid vegetable oil
Margarine	Any solid vegetable fat
Butter	Includes other animal fats, such as lard and tallow
Dressings and sauces	Any kind of salad dressing and sauces high in added sugar, salt, or fats (i.e., thousand islands, catsup)
Candy and sugars	Include sugar, honey, syrup, chocolates, and other confectionaries
Fruit and vegetable juices	Only 100% fruit and vegetable juice
Coffee and tea	Any coffee or tea
Sodas	Include any commercial beverage with added sweeteners
Alcoholic drinks	Any type (beer, wine, spirits, etc.)
Water	Tap or bottled, distilled, or sparkling

This table provides descriptions of the foods included in each food group.

### 2.3 Environmental impact assessment

CFP is chosen as the primary indicator of food's environmental impact, as it is an important indicator at a global level of public concern and the most widely studied environmental metric in most analyses, providing the best data availability for fair and comprehensive comparison across food groups (26–29). Incorporating less often reported environmental indicators, such as biodiversity loss or terrestrial acidification, would result in data gaps across several included food groups. Additionally, foods tend to have similar magnitude environmental impacts across different indicators (20, 30, 31). For example, previous studies regarding meat production support strong correlations between GHG emissions and land use (*r* = 0.67, *P* < 0.05), eutrophication (*r* = 0.88, *P* < 0.05), and acidification (*r* = 0.78, *P* < 0.05) (32). Another comprehensive analysis of CFP also showed significant relationships with eutrophication (*r* = 0.89, *P* < 0.05), acidification (*r* = 0.72, *P* < 0.05), and freshwater aquatic ecotoxicity (*r* = 0.77, *P* < 0.05) (33). Positive correlations between these environmental impacts are to be expected when considering the interactions across farming systems and the environment. For example, land use, including clearing forest for agriculture, directly contributes to CO_2_ emissions. Increased use of fertilizer for intensive farming drives eutrophication potential through agricultural runoff and also is associated with higher CO_2_ emissions from the fertilizer production. Acidification is driven by higher atmospheric CO_2_ (34), which negatively impacts many marine species. Thus, CFP serves as a reasonable proxy for land use, acidification, and eutrophication potentials. One limitation of this approach is that specific environmental impacts, such as water consumption or toxic substance emissions, do not correlate well with CFP (35, 36).

Environmental impact assessments used in this analysis are based on serving sizes of ready to eat foods, reflecting the typical amount of food consumed per occasion (37). Serving sizes are defined using reference amounts customarily consumed established by the U.S. Food and Drug Administration to facilitate comparison across different food types (38). To ensure consistency, the serving sizes for all grains match the serving size for refined grains.

Environmental impacts of food products are evaluated through studies using the life cycle assessment (LCA) methodology (39). To ensure reliable and consistent results, LCA meta-analyses providing the CFP of individual foods and food groups are used (40–43). In cases of missing information, additional systematic reviews and individual LCAs provide supplemental data. The complete list of foods' CFP and the studies used are provided in Supplementary Table S1. Studies including cradle to factory gate system boundaries are prioritized to balance consistency of results reported and availability of data. Although more comprehensive, cradle to grave LCA data is less commonly available for foods and results can be significantly affected by differing assumptions for use phase and disposal. Additionally, the proportion of total impacts from the cradle to gate phase often significantly exceeds the proportion of total impacts from the gate to grave phase (40).

The CFP of each food group is calculated as the average of individual CFP values associated with foods extracted from multiple sources. Meta-analyses are given higher weight in the calculation of food group CFP by treating them as equal to the average of the sum of the individual food CFP values because they are considered “representative” of that group. For example, for the “vegetables” group in Supplementary Table S1, “vegetables (field grown)” is based on a meta-analysis with an extensive database; hence, the calculated mean value of 43 g CO_2_ [=0.5 kg CO_2_ eq/kg × 85 g (serving size)] is given a higher weight relative to the individual food items in that group such as tomatoes. The calculation of the food group average for this category is as shown below.


$$\begin{array}{l} \text{ Vegetables CFP in g of C}{\text{O}}_{2}\text{ eq}/\text{serving}\\ =\frac{\left(85^{*}0.5\right)+\left(85^{*}\frac{2.1+0.5+0.5+0.5}{4}\right)}{2}=60\; \end{array}$$

Food groups are then classified based on their average CFP per serving as low impact (< 100 g of CO_2_-eq/serving), Medium impact (between 100 and 300 g of CO_2_-eq/serving inclusive), and high impact (more than 300 g of CO_2_-eq/serving). This approach of averaging values from various studies may introduce some unavoidable random errors in the final average. The choice of cutoff points is described in the Thresholds and The Matrix Display section below.

### 2.4 The health index score (HIS)

Health impacts of foods are determined by evaluating associations between food intake and health outcomes including the most common chronic diseases such as Type 2 Diabetes Mellitus (T2DM), Coronary Heart Disease (CHD), Cardiovascular Disease (CVD), Colorectal Cancer (CRC), and stroke, as well as All-Cause Mortality (ACM). Evaluation is based on the results from epidemiological research of dose-response analysis focusing on the health impact of consuming an additional serving of a food per day. Where possible, health effects are matched with serving sizes used to assess the CFP to maintain consistency. Priority is given to meta-analyses and systematic reviews of prospective cohort studies and controlled trials rather than individual studies to minimize the potential for biased conclusions.

Extensive research documents the association between food groups and disease risk using relative risk (RR) values. To reflect the overall impact of these risks, we created a unified Health Index Score (HIS), which accounts for the combined RR for ACM, CHD, CVD, CRC, T2DM, and stroke. The HIS was calculated as a weighted average, with the RR of CHD, CVD, T2DM, cancer, and stroke averaged first, followed by incorporating the ACM RR value when data was available to give a greater weight to the ACM:


$$\begin{array}{l} HIS=\frac{RR_{ACM}+\frac{{\sum_{i=1}^{n}RR}_{i}}{n}}{2}\; \end{array}$$

where *RR*_*ACM*_ is the RR for ACM, and *RR*_*i*_ is the RR of the *n* reported diseases. The HIS values classified food groups into three categories: favorable (HIS < 0.96), unfavorable (HIS > 1.07), and neutral (0.96 ≤ HIS ≤ 1.07).

Twenty-one out of the 30 food groups have sufficient information available regarding their impact on health (Figure 3) to calculate *HIS*. Supplementary Table S2 provides the details of information used in calculating HIS, including RR for different health outcomes at indicated consumption levels.

Some food groups do not have a single RR value due to the diversity of constituent foods or due to a lack of available literature. These food groups included savory snacks, dressings and sauces, candy and sugar, ready-to-eat cereals, and pastries and desserts. The health impact of these foods is assessed based on the RR values or other health outcomes associated with their main ingredients of concern, such as added sugar, sodium, and trans fats, which is summarized in Supplementary Table S3. The same classification criteria used for the HIS are applied to the RR values of these ingredients. Given that all these ingredients had RR values >1.07 for various chronic diseases (except for trans fats concerning ischemic stroke, where the RR = 1.07), these food groups are deemed to have adverse health effects if they contain substantial amounts of the mentioned ingredients. Limited data are available on the health effects of consuming shellfish, and no information is available regarding its association with ACM. The only systematic review found on shellfish consumption is limited in scope (44). Despite conflicting opinions regarding the health risks of alcohol consumption, the recommendation of the 2020 Dietary Guidelines Advisory Committee report discourages its consumption and that is reflected in its representation here (45, 46).

### 2.5 Thresholds and the matrix display

The HIS and CFP thresholds in this study are carefully selected to align with the available knowledge derived from prior publications (18, 20, 27, 30, 47–49). This is achieved through a reverse process of using “what is known” (i.e., the impact of the food groups on human health and the environment) to estimate “what is unknown” (i.e., the thresholds).

**Figure 1:**
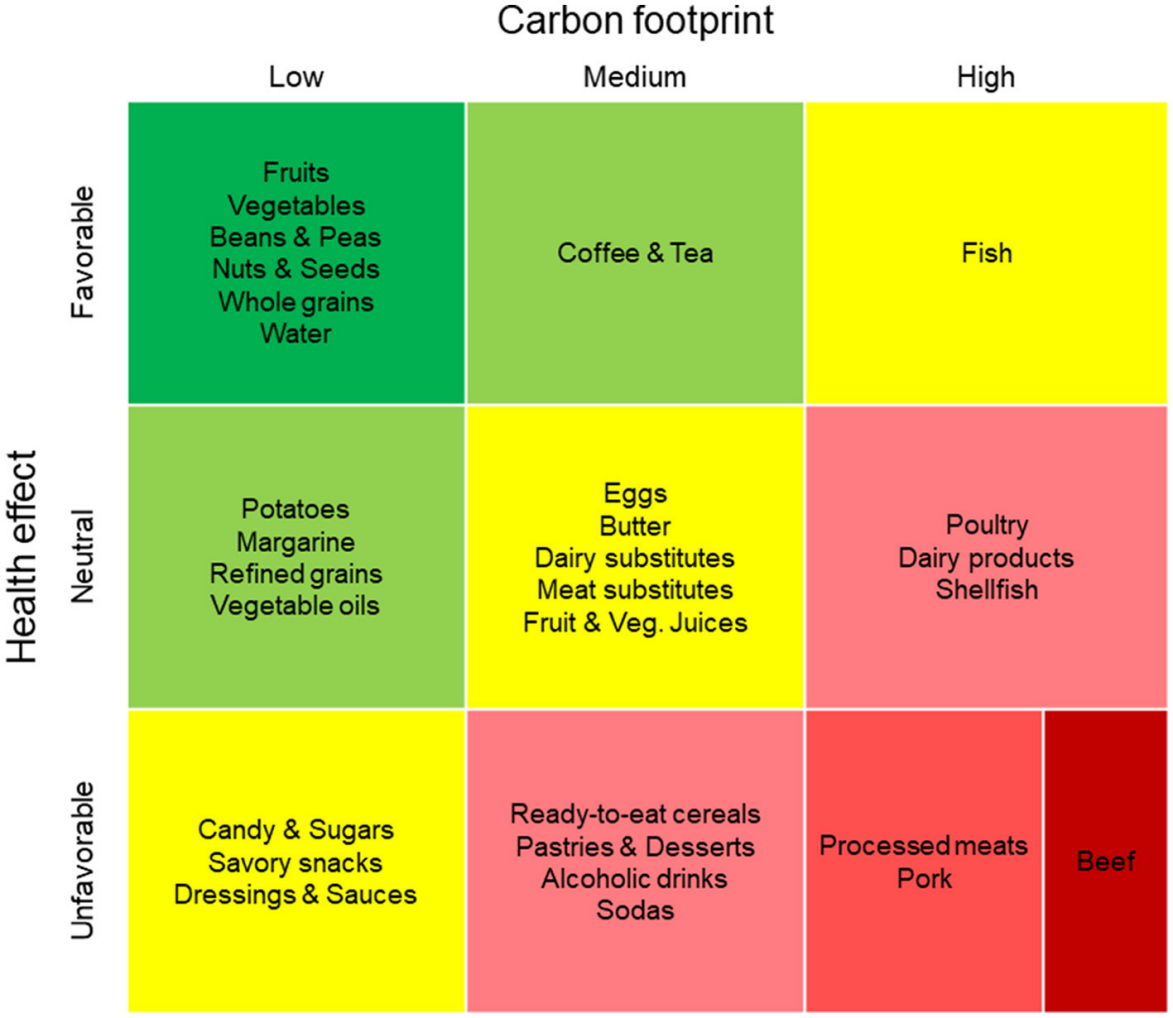
A matrix visualization displaying health effects and carbon footprints for 30 food groups. Groups are arranged in the matrix from top (favorable) to bottom (unfavorable) and from left (low carbon) to right (high carbon). Beef is split vertically in the bottom-right to show its exceptionally high carbon footprint. Cell colors utilize a traffic light scheme to correspond to intensity of environmental and health impacts.

The 30 food groups are organized into a two-dimensional 3 × 3 matrix (Figure 1) to provide an easy-to-compare visual representation of the health and environmental data. This format allows for a clear comparison of different food groups' health and environmental impacts as well as their tradeoffs. Food groups are categorized into nine matrix cells based on their estimated CFP per serving (low, medium, or high) and associated health effects (favorable, neutral, or unfavorable). The food categories are arranged in ascending order of their CFP per serving from left to right along the *x*-axis and from a favorable to an unfavorable overall health effect descending along the *y*-axis. In addition, a color code is applied to intuitively convey the health and environmental impacts of different food groups.

## 3 Results

### 3.1 Carbon footprint of food groups

**Figure 2:**
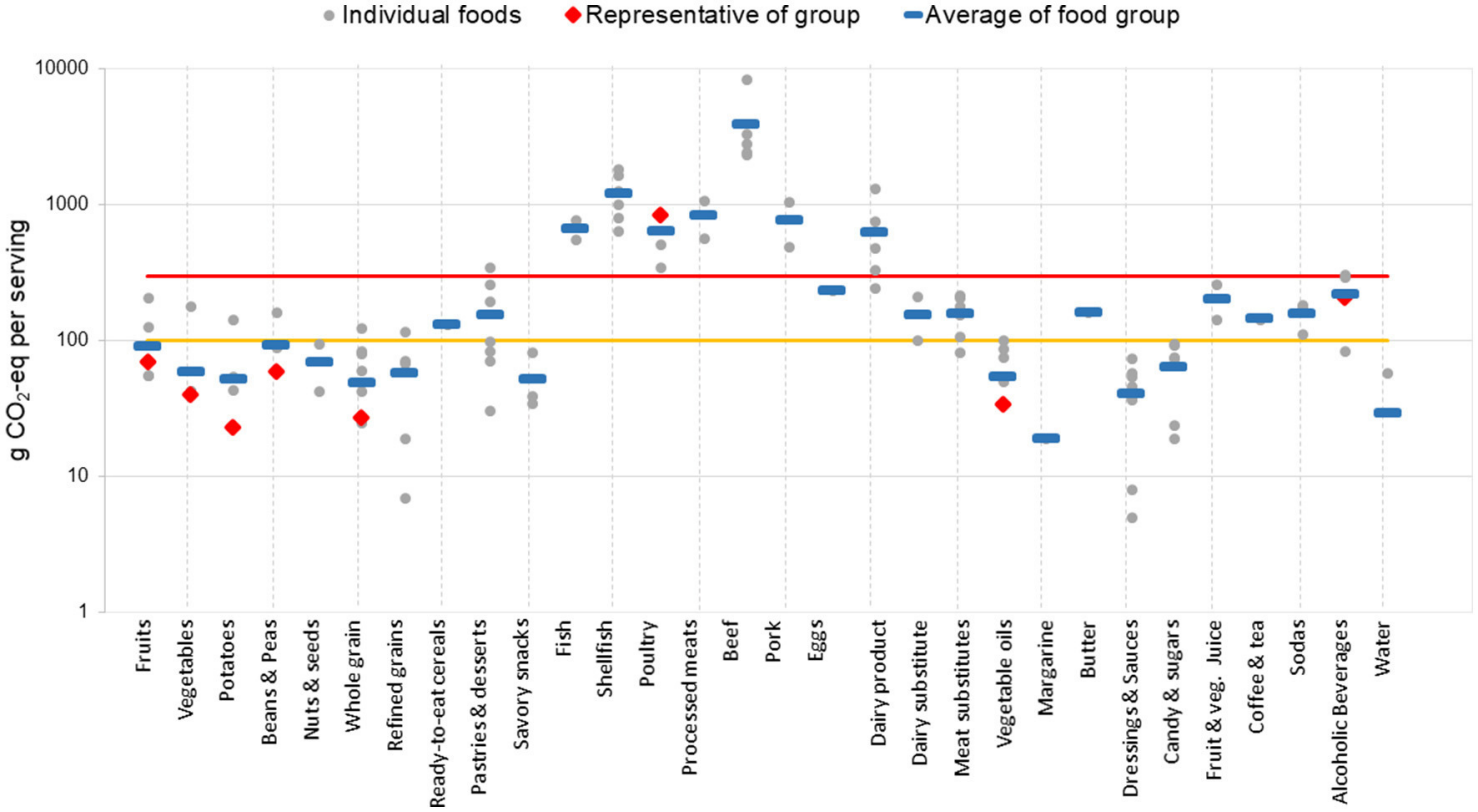
Carbon footprints per serving for 30 food groups, classified as low, medium, or high carbon. Below the yellow line is low, above the red line is high, and in between the yellow and red lines is medium. Blue lines indicate food group averages, used to determine impact classification. A logarithmic scale is utilized on the y-axis.

A substantial variation in CFP among the 30 food groups is observed, with mean values ranging from a low of 20 g CO_2_-eq per serving for Margarine to a high of 3,895 g CO_2_-eq per serving for Beef (Figure 2). Note the logarithmic *y*-axis for CFP per serving.

Food groups with a low CFP (< 100 g CO_2_-eq) include whole plant foods such as fruits, vegetables, potatoes, beans and peas, nuts and seeds, and whole grains, as well as some processed foods like refined grains, savory snacks, vegetable oils, margarine, dressings and sauces, candy and sugars, and water. In the medium CFP group (100 ≤ g CO_2_-eq per serving ≤ 300), there are animal foods such as eggs and butter, processed foods such as ready-to-eat-cereals, pastries and desserts, dairy substitutes, meat substitutes, and fruit and vegetable juices, as well as coffee and tea, sodas, and alcoholic drinks. The high CFP group (g CO_2_-eq per serving >300) consists of animal products, including fish (farmed and wild-caught), shellfish, poultry, processed meats, beef, pork, and dairy. The CFPs of wild-caught and farmed fish are similar (Supplementary Table S1), although there is a substantial difference between fish and shellfish.

### 3.2 Health effects of food groups

**Figure 3:**
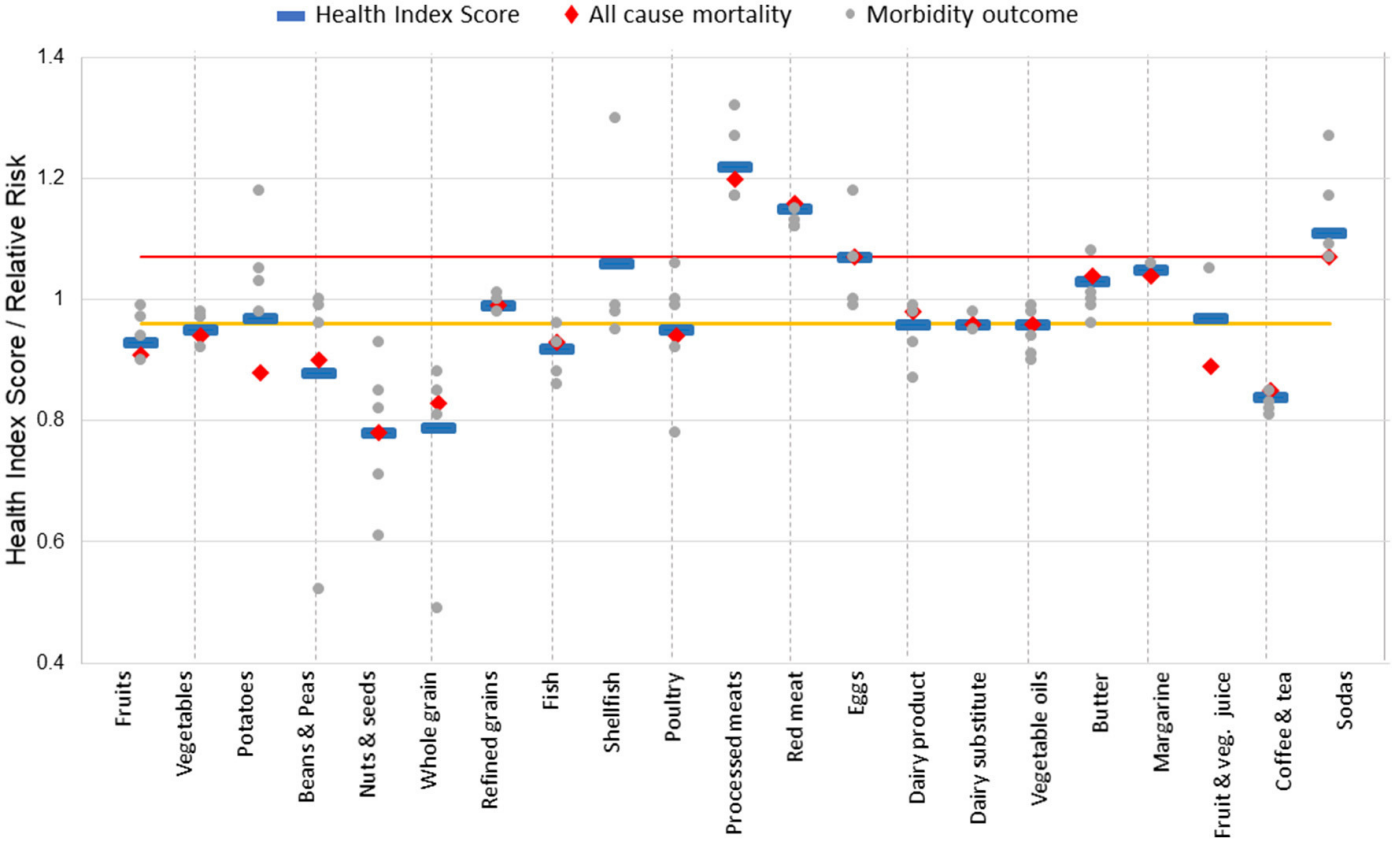
Health effects for 21 food groups, classified as favorable, neutral, and unfavorable. Below the yellow line is favorable, above the red line is unfavorable, and in between the yellow and red lines is neutral. Blue lines indicate Health Index Scores, used to determine health classification.

There is substantial variation in HIS among the food groups compared, with a wide range from the most favorable at 0.77 for nuts and seeds to the least favorable at 1.22 for processed meats (Figure 3).

Fruits, vegetables, beans and peas, nuts and seeds, whole grains, fish, and coffee and tea have favorable effects on human health as they reduce the risk of ACM and/or one or more chronic diseases (HIS < 0.96). Among plant-based foods, potatoes, refined grains, fruit and vegetable juices, dairy substitutes, vegetable oils, and margarine are neutral as they have HIS scores ranging from 0.96 to 1.07. Among animal-based foods, poultry, shellfish, eggs, dairy products, and butter are also considered neutral. Meanwhile, processed and red meats, as well as sodas, which are consistently linked with adverse health effects that increase the risk of ACM and other chronic diseases, are classified in the unfavorable group with HIS > 1.07.

Savory snacks, dressings and sauces, candy and sugar, ready-to-eat cereals, pastries and desserts commonly include excess trans fats, added sugars, and sodium. Consumption of these ingredients is linked to higher mortality rates and increased disease risk (50). Although data on the health effects of consuming such foods is lacking, it is expected that they have an adverse impact on health due to their unhealthy ingredients.

### 3.3 The matrix: combined health and environmental impacts

The comparison of Figures 2, 3 reveals similarities in the degree of health and environmental impacts of certain foods. The 3 × 3 matrix (Figure 1) plots the environmental impact of each food group against their health effects.

The matrix highlights the fact that healthier foods, represented in green, also generally have a lower environmental impact. However, there are some tradeoffs between the two dimensions for certain food groups. For example, fish and some highly processed foods, shown in yellow, present a tradeoff between their health and environmental effects. The red cell containing pork, processed meats, and beef is divided into two sections to reflect the significant range of the CFP within this category. Ruminant meat has an exceptionally high CFP, represented by the darker red color of beef.

## 4 Discussion

### 4.1 Implications

These findings, based on the analysis of the 30 most commonly consumed food groups in terms of their human health and environmental impacts, indicate that foods with favorable health effects are often less impactful on the environment, consistent with academic consensus on the topic (21, 31). In contrast to whole and unrefined plant foods which are more protective of human and planetary health, processed and red meats typically have negative consequences for both. However, selecting foods that benefit both human health and the environment is not always a straightforward decision, and many other factors can play a significant role in food choices.

### 4.2 Methodological decisions

The presented matrix is an effort to provide a simple visual understanding of the relationship between foods' CFP and health implications and highlight their tradeoffs to help inform such decisions. The assessment is based on serving size rather than a standard weight such as 1 kg or specific or aggregate nutrient content, as this allows for a more direct comparison of environmental and health outcomes, provides a reasonable estimate of average consumption, and corresponds to more available data in the literature. Despite being major factors influencing decision-making, cost and taste preferences are not included in the visualization due to their substantial variation among different food groups and across various stakeholders.

### 4.3 Scoring and thresholds compared to existing literature

The HIS introduced in this article is innovative because it combines multiple health outcomes into one score, providing a unified approach to assessing the health effects of food. Additionally, the HIS and CFP thresholds developed in this research could be applied to classify similar or other food groups as well as individual foods in future studies (e.g., applying the matrix approach to foods in a different culture or geographical location). Different approaches to the calculation of HIS and CFP or different thresholds for the classification of the food groups could lead to different outcomes for the final visualization. This potential limitation is addressed through a combination of transparency in methodology and comparison to previous publications to ensure similar outcomes to the established literature. As in all such literature, data availability and quality for both health and environmental implications are a limitation. Life cycle assessments only provide estimates of environmental impacts, and only for specific situations and practices, and health outcomes are based on a wide variety of factors in addition to food choices.

To demonstrate how the thresholds are determined and how they compare to similar studies, a comprehensive study by Clark et al. (20), serves as a reference where three categories of food groups are identified. Those categories include food groups with a RR confidence interval entirely below 1, entirely above 1, or including 1. These three categories correspond to the HIS values of < 0.96 (favorable), >1.07 (unfavorable), and in-between (neutral), respectively. The primary food groups identified in these categories correspond well to those of the present study (Favorable: e.g., whole grains, fruits, vegetables, nuts, fish; Unfavorable: e.g., red meat and processed meat; and Neutral: e.g., potatoes, refined grains, eggs, chicken, dairy).

A comparison can also be drawn between the CFP thresholds in this study and those in the works of Clark et al. (21) and Poore and Nemecek (40), where again, the primary food groups fall into similar categories. According to these studies, high impact food groups, such as beef, pork, cheese, fish, shellfish, poultry, and dairy, match the food groups with the highest GHG emissions in those two studies. Low impact food groups, such as fruits, vegetables, potatoes, nuts, and vegetable oils, also align with food groups with the lowest emissions in those studies. Finally, medium impact food groups, such as eggs, correspond to those with medium emissions in the studies above.

### 4.4 Novelty

This work aims to broaden the scope of nutrition frameworks by including new food groups such as water, fruit and vegetable juices, coffee and tea, alcoholic drinks, dairy substitutes, meat substitutes, shellfish, butter, margarine, vegetable oils, candy and sugars, savory snacks, dressings and sauces, ready-to-eat cereals, and pastries and desserts. While some of these food groups are examined in other studies focusing on GHG emissions (40), their health effect is not typically evaluated. Additionally, while previous research utilizes graphical methods to assess food's health and environmental impact (15, 20, 48, 51), such visualizations can be difficult for non-experts to understand. Therefore, this study presents a comprehensive overview of food choices in an easily accessible format to reach a broader audience. This study focuses on the associations between food and health outcomes rather than solely their nutrient profiles (48, 49). The complexity of nutrient-health associations and the synergistic effects of multiple nutrients make it challenging to predict the health impact of whole foods based on their nutrient profile alone.

### 4.5 Audience

The intended audience for the visualization introduced in this article includes all people seeking to examine potential tradeoffs between health effects and environmental impacts based on broad categories of commonly consumed foods. Such stakeholders include consumers, health professionals, businesses, and policymakers. Some potential applications include helping plan healthier and more sustainable meals and grocery lists, improving the ingredients in manufactured foods, and understanding potential impacts of promoting certain food groups over others. The visualization is designed with a broad, non-expert audience in mind but is sufficiently comprehensive and supported by evidence to support further academic research in this area. The limitations of the matrix, as well as the need for further research, are acknowledged.

### 4.6 Limitations

The visualization is not free of limitations. While using CFP as an indicator of environmental impact is strongly correlated with eutrophication and acidification potentials and land use, it may not accurately reflect the impact on water use, biodiversity, and toxic substance emissions. For instance, some California-grown nuts have a high water demand (52) but a low CFP (53). Incorporating more comprehensive indicators could overcome this limitation if data becomes available for all food groups considered. However, incorporating multiple environmental impacts in one score would require decisions regarding how they are aggregated (e.g., weighting of individual impacts) which introduces additional subjectivity.

Food groups within each matrix cell are presented as equally preferable but are not necessarily equivalent in their environmental and health impacts, and substantial variability exists within some food groups. For instance, virgin olive oil and refined vegetable oils have different health impacts (54), and greenhouse-grown fruits or vegetables have a higher CFP than those grown in fields (42). The disparity in CFP among meats is partially represented, with ruminant meats such as beef having a much higher CFP compared to other meats (40, 42). In addition, the cooking and processing methods used for final preparation of foods can have a significant effect on the health and environmental impacts of the food. However, limited data is available for these potential permutations and representing them would require a far more complex or cluttered visualization. Ultimately, creating a simple, categorical matrix display requires making tradeoffs between precision and practicality.

Certain foods' health and environmental impacts remain disputed and views regarding their health and sustainability may change as new research emerges. The food groups themselves reflect USA consumption patterns, which may vary substantially from other cultures and geographies, limiting generalizability. The classifications of food groups in this study, based on CFP and estimated health outcomes, may differ from those based on nutrient profiles, single score environmental characterization metrics incorporating multiple types of impacts, or alternative evaluation approaches.

The choice of a functional unit, such as protein quality vs. serving size, can also have an impact the environmental classification of foods (55). As a result, it is important to consider multiple sources and perspectives when evaluating the impacts of different food choices. It is worth noting that the food's life cycle goes beyond our project's system boundaries, which can affect estimated CFP. For instance, transportation methods and distance play a role in determining the total CFP of food (56). However, on average, the impact of transportation is relatively small, accounting for only 4.8% of the total CFP, compared to the much larger impact of land use and agricultural production at 71% (57).

It is also important to keep in mind that the same food can have varying health and environmental consequences based on how it is produced, processed, prepared, and consumed. While many plant-based foods generally exhibit favorable health effects and have low CFP, the degree of processing can alter this relationship (e.g., boiled potatoes are considered healthier than deep fried potato chips despite both being made with potatoes).

## Data Availability

The original contributions presented in the study are included in the article/Supplementary material, further inquiries can be directed to the corresponding author.
