# Supplementary material for: A matrix approach to visually communicate simultaneously the environmental and health impacts of foods
Source: Front Nutr. 2025 Aug 5;12:1572297. doi: 10.3389/fnut.2025.1572297 (PMC12361189; doi:10.3389/fnut.2025.1572297)
Supplement: Supplementary file 1 [file Table_1.docx]

Supplementary Material

# Supplementary Tables Captions

**Supplementary Table 1.** Carbon footprints of individual foods and their food groups. This table displays the carbon footprint of different foods and the means of their groups. It also provides serving size, carbon footprint per serving, and the sources used.

**Supplementary Table 2.** Association between food groups and various health outcomes. This table provides information used in calculating the Health Index Score (HIS), including relative risk (RR) for all-cause mortality and morbidity outcomes and the sources used.

**Supplementary Table 3.** This table presents associations between ingredients of concern in certain food groups that lack relative risk (RR) data and their related health outcomes. These food groups include Ready-to-eat cereals, Pastries & Desserts, Savory snacks, Dressings & Sauces, and Candy & Sugar.

# Supplementary Tables

**Supplementary Table 1.** Carbon footprints of individual foods and their food groups. This table displays the carbon footprint of different foods and the means of their groups. It also provides serving size, carbon footprint per serving, and the sources used.

| **Food Group** | **Foods** | **Kg CO_2_ eq/kg**  **product** | **Serving size** | **Mean g CO_2_** | **Food group**  **g CO_2_** | **R** | |  |  |
| --- | --- | --- | --- | --- | --- | --- | --- | --- | --- |
| Fruits | Fruits (field grown)^1^ | 0.50 (0.1-1.8)^a^ | 140 g | **70** | 91 | L | |  |  |
|  | Citrus fruit^2^ | 0.4 (0.0-0.7)^b^ | 140 g | 56 |  |  |  |  |  |
|  | Bananas^2^ | 0.9 (0.6-1.3)^b^ | 140 g | 126 |  |  |  |  |  |
|  | Apples^2^ | 0.4 (0.3-0.6)^b^ | 140 g | 56 |  |  |  |  |  |
|  | Berries, grapes^2^ | 1.5 (0.6-2.9)^b^ | 140 g | 210 |  |  |  |  |  |
| Vegetables | Vegetables (field grown)^1^ | 0.5 (0.04-2.5)^a^ | 85 g | **43** | 60 | L | |  |  |
|  | Tomatoes^2^ | 2.1 (0.4-12.6)^b^ | 85 g | 179 |  |  |  |  |  |
|  | Onions & leeks^2^ | 0.5 (0.3-0.8)^b^ | 85 g | 43 |  |  |  |  |  |
|  | Brassicas^2^ | 0.5 (0.2-1.2)^b^ | 85 g | 43 |  |  |  |  |  |
|  | Other vegetables^2^ | 0.5 (0.2-1.1)^b^ | 85 g | 43 |  |  |  |  |  |
| Potatoes | Bulbs, roots, and tubers^1^ | 0.2 (0.04-0.6)^a^ | 110 g | **22** | 51 | L | |  |  |
|  | Potatoes^2^ | 0.5 (0.1-0.7)^b^ | 110 g | 55 |  |  |  |  |  |
|  | Cassava^2^ | 1.3 (0.3-2.2)^b^ | 110 g | 143 |  |  |  |  |  |
|  | Root vegetables^2^ | 0.4 (0.2-0.6)^b^ | 110 g | 44 |  |  |  |  |  |
| Beans & Peas | Legumes and pulses^1^ | 0.7 (0.15-2.5)^a^ | 90 g | **63** | 95 | L | |  |  |
|  | Pulses^2^ | 1.8 (0.9-4.0)^b^ | 90 g | 162 |  |  |  |  |  |
|  | Peas^2^ | 1.0 (0.5-1.9)^b^ | 90 g | 90 |  |  |  |  |  |
| Nuts & Seeds | Tree nuts combined^1^ | 1.4 (0.43-3.8)^a^ | 30 g | 42 | 69 | L | |  |  |
|  | Groundnuts^2^ | 3.2 (1.4-6.2)^b^ | 30 g | 96 |  |  |  |  |  |
| Whole grains | Cereals^1^ | 0.5 (0.1-1.4)^a^ | ~50 g | **25** | 72 | L | |  |  |
|  | Rice^2^ | 2.7 (0.6-5.7)^e^ | ~50 g | 135 |  |  |  |  |  |
|  | Rice^2^ | 4.5 (1.2-10.3)^e^ | ~50 g | 225 |  |  |  |  |  |
|  | Wheat & rye^2^ | 1.6 (0.7-3.1)^b^ | ~50 g | 80 |  |  |  |  |  |
|  | Cornmeal^2^ | 1.7 (0.7-3.5)^b^ | ~50 g | 85 |  |  |  |  |  |
|  | Barley^2^ | 1.2 (0.6-1.8)^b^ | ~50 g | 60 |  |  |  |  |  |
|  | Oatmeal^2^ | 2.5 (0.8-4.3)^b^ | ~50 g | 125 |  |  |  |  |  |
| Refined grains | White bread^3^ | 2.3^d^ | 50 g | 115 | 59 | L | |  |  |
|  | White rolls^3^ | 1.2^d^ | 50 g | 60 |  |  |  |  |  |
|  | White bread^3^ | 1.4^d^ | 50 g | 70 |  |  |  |  |  |
|  | Cornbread^3^ | 1.4^d^ | ~50 g | 70 |  |  |  |  |  |
|  | White pasta^3^ | 0.4^d,e^ | ~50 g | 20 |  |  |  |  |  |
|  | White rice^3^ | 0.4^d,e^ | ~50 g | 20 |  |  |  |  |  |
| Ready-to-eat cereals | Breakfast cereal^4^ | 2.6^d^ | 50 g | 132 | 130 | M | |  |  |
| Pastries & Desserts | | Cakes and pastry^5^ | 3.3^d^ | 80 g | 266 | 157 | M | | |
|  |  | Sweet rolls and brioches^5^ | 0.6^d^ | 55 g | 33 |  |  |  |  |
|  |  | Gingerbread cake^6^ | 1.2 (1.2-1.6)^a^ | 80 g | 96 |  |  |  |  |
|  |  | Small cookies^6^ | 2.4 (0.87-5)^a^ | 30 g | 72 |  |  |  |  |
|  |  | Large cookies^6^ | 2.8 (1.12-5.38)^a^ | 30 g | 84 |  |  |  |  |
|  |  | Croissants^6^ | 3.6 (3.1-4.3)^a^ | 55 g | 198 |  |  |  |  |
|  |  | Pastries^6^ | 2.8 (1.1-5.4)^a^ | 125 g | 350 |  |  |  |  |
| Savory snacks | | Salty snacks^6^ | 2.8 (1.0-7.1)^a^ | 30 g | 84 | 50 | L | | |
|  |  | Crackers^6^ | 1.3 (1-2.7)^a^ | 30 g | **39** |  |  |  |  |
|  |  | Chips, snacks^6^ | 1.2^d^ | 30 g | 36 |  |  |  |  |
| Fish | | Farmed all^7^ | 6.5 (5.7-7.5)^c^ | 85 g | 553 | 663 | H | | |
|  |  | Wild all^7^ | 9.1 (5.9-13.3)^c^ | 85 g | 774 |  |  |  |  |
| Shellfish | | Prawns/shrimp^1^ | 14.9 (5.3-4)^a^ | 85 g | 1267 | 1256 | H | | |
|  |  | Lobster^1^ | 21.7 (7.6-28.3)^a^ | 85 g | 1845 |  |  |  |  |
|  |  | Shrimp farmed^7^ | 9.4 (8.8-10.1)^c^ | 85 g | 799 |  |  |  |  |
|  |  | Shrimp wild^7^ | 12 (9.7-14.3)^c^ | 85 g | 1020 |  |  |  |  |
|  |  | Lobster wild^7^ | 19.4 (16.1-23.1)^c^ | 85 g | 1649 |  |  |  |  |
| Poultry | | Poultry^2^ | 9.9 (4.0-20.8)^a^ | 85 g | **842** | 636 | H | | |
|  |  | Chicken^1^ | 4.1 (1.1-10.0)^a^ | 85 g | 349 |  |  |  |  |
|  |  | Turkey^1^ | 6.0 (3.3-8.5)^a^ | 85 g | 510 |  |  |  |  |
| Processed meats | | Processed meat^6^ | 19.8 (4.4-79.2)^a^ | 55 g | 1089 | 828 | H | | |
|  |  | Cold cuts^6^ | 10.3 (4.4-23.8)^a^ | 55 g | 567 |  |  |  |  |
| Beef | | Beef: world average^1^ | 28.7 (10.7-109)^a^ | 85 g | 2440 | 3895 | H | | |
|  |  | Lamb: world average^1^ | 27.9 (10.1-56.7)^a^ | 85 g | 2372 |  |  |  |  |
|  |  | Bovine meat (beef herd) ^2^ | 99.5 (37.6-269.2)^a^ | 85 g | 8458 |  |  |  |  |
|  |  | Bovine meat (dairy herd) ^2^ | 33.3 (14.9-56.7)^a^ | 85 g | 2831 |  |  |  |  |
|  |  | Lamb & mutton^2^ | 39.7 (23.7-60.2)^a^ | 85 g | 3375 |  |  |  |  |
| Pork | | Pork^1^ | 5.9 (3.2-11.9)^a^ | 85 g | 502 | 774 | H | | |
|  |  | Pig meat^2^ | 12.3 (6.9-23.8)^a^ | 85 g | 1046 |  |  |  |  |
| Eggs | | Eggs^2^ | 4.7 (2.9-8.5)^b^ | 50 g | 235 | 235 | M | | |
| Dairy products | | | Milk^1^ | 1.4 (0.5-7.5)^a^ | 240 mL | 336 | 483 | H |  |
|  |  |  | Yogurt^1^ | 1.4 (1.2-2)^a^ | 170 g | **238** |  |  |  |
|  |  |  | Cheese^1^ | 8.9 (5.3-16.3)^a^ | 55 g | 490 |  |  |  |
|  |  |  | Milk^2^ | 3.2 (1.5-7.0)^a^ | 240 mL | 768 |  |  |  |
|  |  |  | Cheese^2^ | 23.9 (10.2-58.8)^a^ | 55 g | 1315 |  |  |  |
| Dairy substitutes | | | Almond, coconut milk^1^ | 0.42 (0.39-0.44)^a^ | 240 mL | 101 | 156 | M |  |
|  |  |  | Soy milk^1^ | 0.9 (0.7-1.4)^a^ | 240 mL | 216 |  |  |  |
| Meat substitutes | | | Tofu^8^ | 1.0 (0.9-1.1)^b^ | 85 g | **85** | 129 | M |  |
|  |  |  | Plant protein burger^9^ | 2.5 (0.9-3.7)^a^ | 85 g | 213 |  |  |  |
|  |  |  | Plant protein sausages^9^ | 2.5 (1.3-4.2)^a^ | 85 g | 213 |  |  |  |
|  |  |  | Plant protein nuggets^9^ | 2.1 (1.2-3.4)^a^ | 85 g | 179 |  |  |  |
|  |  |  | Plant protein cold cuts^9^ | 2.0 (1.7-2.3)^a^ | 55 g | 110 |  |  |  |
|  |  |  | Plant protein ground^9^ | 1.8 (1.3-2.4)^a^ | 85 g | 153 |  |  |  |
| Vegetable oils | | | Cooking oils and fats^6^ | 2.4 (1.9-2.9)^a^ | 14 g | **34** | 54 | L |  |
|  |  |  | Soybean oil^2^ | 6.3 (2.2-18.8)^a^ | 14 g | 88 |  |  |  |
|  |  |  | Palm oil^2^ | 7.3 (2.8-13.1)^a^ | 14 g | 102 |  |  |  |
|  |  |  | Sunflower oil^2^ | 3.6 (2.2-4.9)^a^ | 14 g | 50 |  |  |  |
|  |  |  | Canola oil^2^ | 3.8 (2.2-7.2)^a^ | 14 g | 53 |  |  |  |
|  |  |  | Olive oil^2^ | 5.4 (2.1-10.8)^a^ | 14 g | 76 |  |  |  |
| Margarine | | | Margarine^10^ | 1.4 (1.1-1.7)^a^ | 14 g | 20 | 20 | L |  |
| Butter | | | Butter^1^ | 11.5 (3.7-25)^a^ | 14 g | 161 | 161 | M |  |
| Dressings & Sauces | | | Tomato sauce^3^ | 0.9^d^ | 60 g | 54 | 40 | L |  |
|  |  |  | Catsup/ketchup^3^ | 0.5^d^ | 15 g | 8 |  |  |  |
|  |  |  | Thousand Island dressing^3^ | 1.9^d^ | 30 g | 57 |  |  |  |
|  |  |  | Ranch dressing^3^ | 2.5^d^ | 30 g | 75 |  |  |  |
|  |  |  | Italian dressing^3^ | 1.2^d^ | 30 g | 36 |  |  |  |
|  |  |  | Low-calorie dressing^3^ | 1.3^d^ | 30 g | 39 |  |  |  |
|  |  |  | Regular mayonnaise^3^ | 3.1^d^ | 15 g | 47 |  |  |  |
|  |  |  | Low-calorie mayonnaise^3^ | 0.30^d^ | 15 g | 5 |  |  |  |
| Candy & Sugars | | | Chocolate and chocolate products^5^ | 3.1^c,d^ | 30 g | 93 | 64 | L |  |
|  | Sugars, honey, syrups^5^ | 0.9^c,d^ | 21 g | 19 |  |  | |  |  |
|  | Non-chocolate candy^5^ | 2.5^c.d^ | 30 g | 75 |  |  | |  |  |
|  | Chocolate^6^ | 3.2 (2-4)^a^ | 30 g | 96 |  |  | |  |  |
|  | Candy | 0.8 (0.6-4.73)^a^ | 30 g | 24 |  |  | |  |  |
|  | Sugar and sweets^11^ | 2.5^d^ | 30 g | 75 |  |  | |  |  |
| Fruit & Vegetable juices | Fruit juices^11^ | 0.6^d^ | 240 mL | 144 | 204 | M | |  |  |
|  | Fruit juices^6^ | 1.1 (0.3-4.7)^a^ | 240 mL | 264 |  |  | |  |  |
| Coffee & Tea | Coffee and tea^11^ | 0.4^d^ | 360 mL | 144 | 144 | M | |  |  |
| Sodas | Soft drinks^11^ | 0.5^d^ | 360 mL | 180 | 156 | M | |  |  |
|  | Soft drinks^6^ | 0.5 (0.2-2.8)^a^ | 360 mL | 180 |  |  | |  |  |
|  | Soft drinks^12^ | 0.3 (0.2-0.6)^a^ | 360 mL | 108 |  |  | |  |  |
| Alcoholic drinks | Alcoholic drinks^11^ | 1.1^d^ | 191 mL | **209** | 217 | M | |  |  |
|  | Liqueur^6^ | 1.9 (1.6-2.6)^a^ | 43 g | 82 |  |  | |  |  |
|  | Bee^6^ | 0.8 (0.7-0.9)^a^ | 360 mL | 288 |  |  | |  |  |
|  | Wine^2^ | 1.8 (0.7-4.7)^a^ | ~170 mL | 306 |  |  | |  |  |
| Water | Tap water^13^ | 0.001^d^ | 360 mL | 0.4 | 29 | L | |  |  |
|  | Bottled water^13^ | 0.16^d^ | 360 mL | 58 |  |  | |  |  |

CO_2_-eq = Kg of Carbon dioxide equivalents per Kg of product

R = Rating: L=low, M=medium, H=high

a = Mean (Min-Max)

b = 5-95 percentile

c = Median

d = Data for min and max/confidence interval not reported.

e = To account for expansion in cooking, CO2-eq per serving is divided by 5.26 for dry rice and 2.42 for dry pasta.

f = The serving size used differs from the RACC recommended value and was adjusted for consistency with comparison foods.

**Bold numbers** = Bold items are based on large meta-analysis databases for a food group rather than individual foods and are considered the group representative. Therefore, they are given a larger weight relative to the individual items of the group. This is accomplished by first averaging the individual items and then taking the average of that and the representative group values.

**Supplementary Table 2**. Association between food groups and various health outcomes. This table provides information used in calculating the Health Index Score (HIS), including relative risk (RR) for all-cause mortality and morbidity outcomes and the sources used.

| **Food group** | **Study design** | **Consumption level** | **Health result** | **RR** | **CI low** | **CI high** | **HIS** | **R** | | |
| --- | --- | --- | --- | --- | --- | --- | --- | --- | --- | --- |
| Fruits | Meta | 160 g/day | ACM^14^ | **0.91** | 0.89 | 0.93 | 0.93 | F | | |
|  |  | 100 g/day | CHD^15^ | 0.94 | 0.9 | 0.97 |  |  |  |  |
|  |  |  | CRC^14^ | 0.97 | 0.95 | 0.99 |  |  |  |  |
|  |  |  | T2DM^16^ | 0.99 | 0.97 | 1 |  |  |  |  |
|  |  |  | Stroke^15^ | 0.9 | 0.84 | 0.97 |  |  |  |  |
| Vegetables | Meta | 80 g/day | ACM^14^ | **0.94** | 0.93 | 0.96 | 0.95 | F | | |
|  |  | 100 g/day | CHD^15^ | 0.97 | 0.96 | 0.99 |  |  |  |  |
|  |  |  | CRC^14^ | 0.97 | 0.96 | 0.98 |  |  |  |  |
|  |  |  | T2D^16^ | 0.98 | 0.95 | 1.01 |  |  |  |  |
|  |  |  | Stroke^15^ | 0.92 | 0.86 | 0.98 |  |  |  |  |
| Potatoes | Meta | 150 g/day | ACM^17^ | **0.88** | 0.69 | 1.12 | 0.97 | N | | |
|  |  |  | CHD^14^ | 1.03 | 0.96 | 1.09 |  |  |  |  |
|  |  |  | CRC^14^ | 1.05 | 0.92 | 1.2 |  |  |  |  |
|  |  |  | T2DM^14^ | 1.18 | 1.1 | 1.27 |  |  |  |  |
|  |  |  | Stroke^14^ | 0.98 | 0.93 | 1.03 |  |  |  |  |
| Beans & peas | Meta | 100 g/day | ACM^14^ | **0.9** | 0.85 | 0.96 | 0.88 | F | | |
|  |  | 50 g/day | CHD^15^ | 0.96 | 0.92 | 1.01 |  |  |  |  |
|  |  |  | CRC^14^ | 0.99 | 0.92 | 1.06 |  |  |  |  |
|  |  |  | T2DM^17^ | 0.52 | 0.16 | 1.76 |  |  |  |  |
|  |  |  | Stroke^15^ | 1.00 | 0.88 | 1.13 |  |  |  |  |
| Nuts & Seeds | Meta | 28 g/day | ACM^18^ | **0.78** | 0.72 | 0.84 | 0.77 | F | | |
|  |  |  | CHD^18^ | 0.71 | 0.63 | 0.8 |  |  |  |  |
|  |  |  | Cancer^18^ | 0.85 | 0.76 | 0.94 |  |  |  |  |
|  |  |  | T2DM^18^ | 0.61 | 0.43 | 0.88 |  |  |  |  |
|  |  |  | Stroke^18^ | 0.93 | 0.83 | 1.05 |  |  |  |  |
| Whole grains | Meta | 30 g/day* | ACM^14^ | **0.83** | 0.77 | 0.90 | 0.79 | F | | |
|  |  |  | CHD^18^ | 0.81 | 0.75 | 0.87 |  |  |  |  |
|  |  |  | Cancer^18^ | 0.85 | 0.8 | 0.91 |  |  |  |  |
|  |  |  | T2DM^18^ | 0.49 | 0.23 | 1.05 |  |  |  |  |
|  |  |  | Stroke^18^ | 0.88 | 0.75 | 1.03 |  |  |  |  |
| Refined grains | Meta | 30 g/day | ACM^14^ | **0.99** | 0.97 | 1.01 | 0.99 | N | | |
|  |  |  | CHD^15^ | 1.01 | 0.99 | 1.04 |  |  |  |  |
|  |  |  | T2DM^16^ | 0.98 | 0.96 | 1.01 |  |  |  |  |
|  |  |  | Stroke^14^ | 1.00 | 0.98 | 1.01 |  |  |  |  |
| Fish | Meta | 100 g/day | ACM^14^ | **0.93** | 0.88 | 0.98 | 0.92 | F | | |
|  |  | 100 g/day | CHD^15^ | 0.88 | 0.79 | 0.99 |  |  |  |  |
|  |  | 100 g/day | CRC^14^ | 0.93 | 0.85 | 1.01 |  |  |  |  |
|  |  | H-L | T2DM^19^ | 0.96 | 0.85 | 1.1 |  |  |  |  |
|  |  | 100 g/day | Stroke^15^ | 0.86 | 0.75 | 0.99 |  |  |  |  |
| Shellfish | | Cohort | Rarely vs. >1/wk | CHD^20^ | 0.98 | 0.82 | 1.18 | 1.06 | | N |
|  |  | Cohort (W)^a^ | 0 vs. >0.6 g/day | CRC^21^ | 1.30 | 1.00 | 1.60 |  |  |  |
|  |  | Meta | H-L | T2DM^19^ | 0.95 | 0.88 | 1.1 |  |  |  |
|  |  | Systematic Review | Consumers vs. non | Stroke^22^ | 0.99 | 0.73 | 1.37 |  |  |  |
| Poultry | | Meta | H-L | ACM^23^ | **0.94** | 0.9 | 0.97 | 0.97 | | N |
|  |  |  | 100 g/day | CHD^17^ | 1.00 | 0.87 | 1.15 |  |  |  |
|  |  |  |  | CRC^17^ | 0.78 | 0.62 | 0.94 |  |  |  |
|  |  |  |  | T2DM^17^ | 1.06 | 0.81 | 1.35 |  |  |  |
|  |  |  |  | Stroke^24^ | 0.92 | 0.82 | 1.03 |  |  |  |
| Processed meats | | Meta | 60 g/day | ACM^14^ | **1.2** | 1.17 | 1.23 | 1.22 | | U |
|  |  |  | 50 g/day | CHD^15^ | 1.27 | 1.09 | 1.49 |  |  |  |
|  |  |  |  | CRC^14^ | 1.17 | 1.10 | 1.23 |  |  |  |
|  |  |  |  | T2DM^25^ | 1.32 | 1.19 | 1.48 |  |  |  |
|  |  |  |  | Stroke^15^ | 1.17 | 1.02 | 1.34 |  |  |  |
| Red meat (Beef and Pork)^b^ | | Meta | 85 g/day | ACM^14^ | **1.16** | 1.14 | 1.18 | 1.15 | | U |
|  |  |  | 100 g/day | CHD^15^ | 1.15 | 1.08 | 1.23 |  |  |  |
|  |  |  |  | CRC^26^ | 1.12 | 1.06 | 1.19 |  |  |  |
|  |  |  |  | T2DM^25^ | 1.13 | 1.03 | 1.23 |  |  |  |
|  |  |  |  | Stroke^15^ | 1.12 | 1.06 | 1.17 |  |  |  |
| Dairy products | | Meta | 200 g/day | ACM^14^ | **0.98** | 0.93 | 1.03 | 0.96 | | N |
|  |  |  |  | CHD^15^ | 0.99 | 0.96 | 1.02 |  |  |  |
|  |  |  |  | CRC^14^ | 0.93 | 0.91 | 0.94 |  |  |  |
|  |  |  |  | T2DM^18^ | 0.87 | 0.72 | 1.04 |  |  |  |
|  |  |  |  | Stroke^15^ | 0.98 | 0.96 | 1.00 |  |  |  |
| Dairy substitutes | | Meta | High in soy products | ACM^27^ | **0.96** | 0.9 | 1.02 | 0.96 | | N |
|  |  |  |  | CVD^27^ | 0.95 | 0.82 | 1.10 |  |  |  |
|  |  |  |  | Cancer^27^ | 0.98 | 0.92 | 1.05 |  |  |  |
| Vegetable oils | | Meta | 10 g/day  olive oil | ACM^17^ | **0.92** | 0.86 | 0.99 | 0.96 | | N |
|  |  |  |  | CHD^14^ | 0.94 | 0.86 | 1.03 |  |  |  |
|  |  |  |  | T2DM^17^ | 0.91 | 0.87 | 0.95 |  |  |  |
|  |  |  |  | Stroke^17^ | 0.90 | 0.86 | 0.95 |  |  |  |
| Vegetable oils | | Prospective Analysis | 8 g/day  corn oil | ACM^28^ | **0.99** | 0.97 | 1.02 |  |  |  |
|  |  |  |  | CVD+T2DM^28^ | 0.99 | 0.95 | 1.03 |  |  |  |
|  |  |  | 8 g/day  canola oil | ACM^28^ | **0.98** | 0.96 | 1.00 |  |  |  |
|  |  |  |  | CVD+T2DM^28^ | 0.98 | 0.94 | 1.02 |  |  |  |
| Eggs | | Meta | 55 g/day | ACM^14^ | **1.07** | 1.01 | 1.15 | 1.07 | | N |
|  | |  | 50 g/day | CHD^15^ | 1.00 | 0.95 | 1.06 |  | |  |
|  | |  |  | CRC^14^ | 1.18 | 0.89 | 1.56 |  | |  |
|  | |  |  | T2DM^17^ | 1.07 | 0.91 | 1.23 |  | |  |
|  | |  |  | Stroke^15^ | 0.99 | 0.93 | 1.05 |  | |  |
| Butter | | Meta | 14 g/day | ACM^29^ | **1.01** | 1.00 | 1.03 | 1.03 | | N |
|  | |  |  | CVD^29^ | 1.00 | 0.98 | 1.02 |  | |  |
|  | |  |  | CHD^29^ | 0.99 | 0.96 | 1.03 |  | |  |
|  | |  |  | Stroke^29^ | 1.01 | 0.98 | 1.03 |  | |  |
|  | |  |  | T2DM^20^ | 0.96 | 0.93 | 0.99 |  | |  |
| Margarine | | Prospective Analysis | 14 g/day | ACM^30^ | **1.04** | 1.03 | 1.05 | 1.05 | | N |
|  | |  |  | CVD+T2DM^29^ | 1.06 | 1.05 | 1.08 |  | |  |
| Fruit & Vegetable juices | | Prospective Analysis | 250 mL/day | ACM^31^ | **0.89** | 0.81 | 0.97 | 0.97 | | N |
|  | | Meta |  | T2DM^32^ | 1.05 | 0.99 | 1.11 |  | |  |
| Coffee & Tea | | Meta | 3 to 4 coffees /day | ACM^33^ | **0.83** | 0.79 | 0.88 | 0.84 | | F |
|  | |  |  | CVD^33^ | 0.85 | 0.80 | 0.90 |  | |  |
|  | |  | High vs. low | Cancer^33^ | 0.82 | 0.74 | 0.89 |  | |  |
|  | |  | ≥5 cups of green tea /day | ACM^34^ (men) | **0.9** | 0.87 | 0.94 |  | |  |
|  | |  |  | ACM^34^ (women) | **0.82** | 0.74 | 0.9 |  | |  |
| Sodas | | Meta | 225 g/day | ACM^14^ | **1.07** | 1.01 | 1.14 | 1.11 | | U |
|  | |  |  | CHD^17^ | 1.17 | 1.11 | 1.23 |  | |  |
|  | |  |  | CRC^17^ | 1.09 | 0.97 | 1.22 |  | |  |
|  | |  |  | Stroke^17^ | 1.07 | 1.02 | 1.12 |  | |  |
|  | |  |  | T2DM^32^ | 1.27 | 1.10 | 1.46 |  | |  |

Meta= Meta-Analysis

RR= Relative risk; HIS= Health Index Score; R= Rating

N= Neutral; F= Favorable; U=Unfavorabe

CI low= Lower confidence interval; CI high= Higher confidence interval

ACM= All-cause mortality; CVD= Cardiovascular disease; T2DM= Type 2 diabetes mellitus

CHD= Coronary heart disease; CRC= Colorectal cancer

H-L= Highest vs. Lowest

**Bold numbers** = Bold items are based on large meta-analysis databases for a food group rather than individual foods and are considered the group representative. Therefore, they are given a larger weight relative to the individual items of the group. This is accomplished by first averaging the individual items and then taking the average of that and the representative group values.

a= Cohort for women

b= Although we distinguish between beef and pork for environmental impact, meta-analysis sources report RR for red meat as one category

**Supplementary Table** **3.** This table presents associations between ingredients of concern in certain food groups that lack relative risk (RR) data and their related health outcomes. These foods include Ready-to-eat cereals, Pastries & Desserts, Savory Snacks, Dressings & Sauces, and Candy & Sugar

| **Ingredient of Concern** | **Study Design** | **Health outcome** | **Main results** |
| --- | --- | --- | --- |
| Added sugar | Review^35^ | Obesity | Detrimental association |
|  | Meta-analysis^36^ |  | 0.22 kg weight gain per year |
|  | Meta-analysis^37^ | T2DM | RR: 1.25 (1.10 to 1.42) |
|  |  | Metabolic Syndrome | RR: 1.20 (1.02 to 1.42) |
|  | Review^38^ | CVD. T2DM | Detrimental association |
|  |  | Dyslipidemia, insulin resistance, fatty liver | Direct relationship |
| Sodium | Meta-analysis^39^ | Stroke | RR: 1.24 (1.08 to 1.43) |
|  |  | Stroke mortality | RR: 1.63 (1.27 to 2.10) |
|  |  | CHD mortality | RR: 1.32 (1.13 to 1.53) |
| Trans fats | Meta-analysis^40^ | ACM | RR: 1.34 (1.16 to 1.56) |
|  |  | CHD mortality | RR: 1.28 (1.09 to 1.50) |
|  |  | Ischemic stroke | RR: 1.07 (0.88 to 1.28) |
|  |  | T2DM | RR: 1.10 (0.95 to 1.27) |

RR= Relative risk at 95% confidence interval

a= RR and weight gain for added sugar is from 1 additional 12-oz sugar-sweetened per day

ACM= All-cause mortality; CVD= Cardiovascular disease; T2DM= Type 2 diabetes mellitus

CHD= Coronary heart disease

**References**

[1] Clune S, Crossin E, Verghese K. Systematic review of greenhouse gas emissions for different fresh food categories. J Clean Prod. 2017;140:766-83. <https://doi.org/10.1016/j.jclepro.2016.04.082>

[2] Poore J, Nemecek T. Reducing food’s environmental impacts through producers and consumers. Science. 2018;360:987-92. <https://doi.org/10.1126/science.aaq0216>

[3] Berardy A, Fresán U, Matos RA, et al. Environmental impacts of foods in the Adventist health study-2 dietary questionnaire. Sustainability. 2020;12:1-14. <https://doi.org/10.3390/su122410267>

[4] Jeswani HK, Burkinshaw R, Azapagic A. Environmental sustainability issues in the food-energy-water nexus: Breakfast cereals and snacks. Sustain Prod Consum. 2015;2:17-28. <https://doi.org/10.1016/j.spc.2015.08.001>

[5] Drewnowski A, Rehm CD, Martin A, et al. Energy and nutrient density of foods in relation to their carbon footprint. Am J Clin Nutr. 2015;101:184-91. <https://doi.org/10.3945/ajcn.114.092486>

[6] van de Kamp ME, van Dooren C, Hollander A, et al. Healthy diets with reduced environmental impact? – The greenhouse gas emissions of various diets adhering to the Dutch food based dietary guidelines. Food Res Int. 2018;104:14-24. <https://doi.org/10.1016/j.foodres.2017.06.006>

[7] Gephart JA, Henriksson PJG, Parker RWR, et al. Environmental performance of blue foods. Nature. 2021;597:360-365. <https://doi.org/10.1038/s41586-021-03889-2>

[8] Mejia A, Harwatt H, Jacealldo-Siegl K, et al. Greenhouse Gas Emissions Generated by Tofu Production: A Case Study. J Hunger Environ Nutr. 2017;13:131-42. <https://doi.org/10.1080/19320248.2017.1315323>

[9] Mejia MA, Fresán U, Harwatt H, et al. Life cycle assessment of the production of a large variety of meat analogs by three diverse factories. J Hunger Environ Nutr. 2019;0248. <https://doi.org/10.1080/19320248.2019.1595251>

[10] Nilsson K, Flysjö A, Davis J, et al. Comparative life cycle assessment of margarine and butter consumed in the UK, Germany and France. Int J Life Cycle Assess. 2010;15:916-26. <https://doi.org/10.1007/s11367-010-0220-3>

[11] Temme EHM, Toxopeus IB, Kramer GFH, et al. Greenhouse gas emission of diets in the Netherlands and associations with food, energy and macronutrient intakes. Public Health Nutr. 2015;18:2433-2445. <https://doi.org/10.1017/S1368980014002821>

[12] Amienyo D, Gujba H, Stichnothe H, et al. Life cycle environmental impacts of carbonated soft drinks. Int J Life Cycle Asses. 2013;18:77-92. <https://doi.org/10.1007/s11367-012-0459-y>

[13] Fantin V, Scalbi S, Ottaviano G, et al. A method for improving reliability and relevance of LCA reviews: The case of life-cycle greenhouse gas emissions of tap and bottled water. Sci Total Environ. 2014;476-477:228-41. <https://doi.org/10.1016/j.scitotenv.2013.12.115>

[14] Schwingshackl L, Schwedhelm C, Hoffmann G, et al. Food groups and risk of all-cause mortality: a systematic review and meta-analysis of prospective studies. Am J Clin Nutr. 2017;8:793-803. <https://doi.org/10.3945/ajcn.117.153148>

[15] Bechthold A, Boeing H, Schwedhelm C, et al. Food groups and risk of coronary heart disease, stroke and heart failure: A systematic review and dose-response meta-analysis of prospective studies. Crit Rev Food Sci Nutr. 2019;0(0):120. <https://doi.org/10.1080/10408398.2017.1392288>

[16] Wu Y, Zhang D, Hiang X, et al. Fruit and vegetable consumption and risk of type 2 diabetes mellitus: A dose-response meta-analysis of prospective cohort studies. Nutr Metab and Cardiovas. 2015;25(2):140-147. <https://doi.org/10.1016/j.numecd.2014.10.004>

[17] Clark MA, Springmann M, Hill J, et al. Multiple health and environmental impacts of foods. Proc Natl Acad Sci USA. 2019;116:23357-62. <https://doi.org/10.1073/pnas.1906908116>

[18] Aune D. Plant foods, antioxidant biomarkers, and the risk of cardiovascular disease, cancer, and mortality: A Review of the evidence. Adv Nutr. 2019;10:S404-21. <https://doi.org/10.1093/advances/nmz042>

[19] Namazi N, Brett NR, Bellissimo N, at al. The association between types of seafood intake and the risk of type 2 diabetes: A systematic review and meta-analysis of prospective cohort studies. Health Promot Perspect. 2019;9(3):164-173. <https://doi.org/10.15171/hpp.2019.24>

[20] Matheson EM, Mainous AG, Hill EG, et al. Shellfish consumption and risk of coronary heart disease. J Am Diet Assoc. 2009;109(8):1422-1426. <https://doi.org/10.1016/j.jada.2009.05.007>

[21] Lee SA, Shu XO, Yang G, at al. Animal origin foods and colorectal cancer risk: A report from the Shanghai Women’s Health Study. Nutr Cancer. 2009;61(2):194-205. <https://doi.org/10.1080/01635580802419780>

[22] Xu L, Cai J, Gao T, et al. Shellfish consumption and health: A comprehensive review of human studies and recommendations for enhanced public policy. Critic Rev Food Sci Nutr. 2022;62:4656-4668. <https://doi.org/10.1080/10408398.2021.1878098>

[23] Lupoli R, Vitale M, Calabrese I, et al. White meat consumption, all-cause mortality, and cardiovascular events: A meta-analysis of prospective cohort studies. Nutrients. 2021;13:676. <https://doi.org/10.3390/nu13020676>

[24] Mohammadi H, Jayedi A, Ghaedi E, at al. Dietary poultry intake and the risk of stroke: a dose–response meta-analysis of prospective cohort studies. Clin Nutr. 2018;23:25-33. <https://doi.org/10.1016/j.clnesp.2017.11.001>

[25] Feskens EJM, Sluik D, van Woudenbergh GJ. Meat consumption, diabetes, and its complications. Curr Diab Rep. 2013;13:298-306. <https://doi.org/10.1007/s11892-013-0365-0>

[26] FAO. Food-based dietary guidelines. 2022. [Accessed August 3, 2023]. Available from <https://www.fao.org/nutrition/education/food-dietary-guidelines/background/sustainable-dietary-guidelines/en/>.

[27] Namazi N, Saneei P, Larijani B, at al. Soy product consumption and the risk of all-cause, cardiovascular and cancer mortality: A systematic review and meta-analysis of cohort studies. Food Funct. 2018;9:2576-88. <https://doi.org/10.1039/c7fo01622k>

[28] Zhang Y, Zhuang P, Wu F, et al. Cooking oil/fat consumption and deaths from cardiometabolic diseases and other causes: prospective analysis of 521,120 individuals. BMC Med. 2021;19:1-14. <https://doi.org/10.1186/s12916-021-01961-2>

[29] Pimpin L, Wu JHY, Haskelberg H, et al. Is butter back? A systematic review and meta-analysis of butter consumption and risk of cardiovascular disease, diabetes, and total mortality. PLoS One. 2016;11:1-18. <https://doi.org/10.1371/journal.pone.0158118>

[30] Nemecek T, Jungbluth N, Milà I Canals L, et al. Environmental impacts of food consumption and nutrition: where are we and what is next? Int J Life Cycle Assess. 2016;21:607-620. <https://doi.org/10.1007/s11367-016-1071-3>

[31] Anderson JJ, Gray SR, Welsh P, et al. The associations of sugar-sweetened, artificially sweetened and naturally sweet juices with all-cause mortality in 198,285 UK Biobank participants: A prospective cohort study. BMC Med. BMC Medicine. 2020;18:1-12. <https://doi.org/10.1186/s12916-020-01554-5>

[32] Imamura F, O’Connor L, Ye Z, e al. Consumption of sugar sweetened beverages, artificially sweetened beverages, and fruit juice and incidence of type 2 diabetes: Systematic review, meta-analysis, and estimation of population attributable fraction. Br J Sports Med. 2016;50:496-504. <https://doi.org/10.1136/bmj.h3576>

[33] Poole R, Kennedy OJ, Roderick P, at al. Coffee consumption and health: umbrella review of meta-analyses of multiple health outcomes. BMJ. 2017;359:j5024. <https://doi.org/10.1136/bmj.j5024>

[34] Abe SK, Saito E, Sawada N, et al. Green tea consumption and mortality in Japanese men and women: a pooled analysis of eight population-based cohort studies in Japan. Eur J Epidemiol. 2019;34:917-26. <https://doi.org/10.1007/s10654-019-00545-y>

[35] Evans CEL. Sugars and health: A review of current evidence and future policy. Proc Nutr Soc. 2017;76:400-7. <https://doi.org/10.1017/S0029665116002846>

[36] Malik VS, Pan A, Willett WC, et al. Sugar-sweetened beverages and weight gain in children and adults: a systematic review and meta-analysis. MCN Am J Matern Nurs. 2014;39:67. <https://doi.org/10.3945/ajcn.113.058362>

[37] Malik VS, Popkin BM, Bray GA, et al. Sugar-sweetened beverages and risk of metabolic syndrome and type 2 diabetes: A meta-analysis. Diabetes Care. 2010;33:2477-2483. <https://doi.org/10.2337/dc10-1079>

[38] Stanhope K. Sugar consumption, metabolic disease and obesity: The state of the controversy. Crit Rev Clin Lab Sci. 2016;53:52-67. <https://doi.org/10.3109/10408363.2015.1084990>

[39] Aburto NJ, Ziolkovska A, Hooper L, et al. Effect of lower sodium intake on health: Systematic review and meta-analyses. BMJ. 2013;346:1-20. <https://doi.org/10.1136/bmj.f1326>

[40] De Souza RJ, Mente A, Maroleanu A, et al. Intake of saturated and trans unsaturated fatty acids and risk of all-cause mortality, cardiovascular disease, and type 2 diabetes: Systematic review and meta-analysis of observational studies. BMJ. 2015;351:1-16. <https://doi.org/10.1136/bmj.h3978>
